# Mine safety assessment using gray relational analysis and bow tie model

**DOI:** 10.1371/journal.pone.0193576

**Published:** 2018-03-21

**Authors:** Qingwei Xu, Kaili Xu

**Affiliations:** School of Resources and Civil Engineering, Northeastern University, Shenyang, China; Southwest University, CHINA

## Abstract

Mine safety assessment is a precondition for ensuring orderly and safety in production. The main purpose of this study was to prevent mine accidents more effectively by proposing a composite risk analysis model. First, the weights of the assessment indicators were determined by the revised integrated weight method, in which the objective weights were determined by a variation coefficient method and the subjective weights determined by the Delphi method. A new formula was then adopted to calculate the integrated weights based on the subjective and objective weights. Second, after the assessment indicator weights were determined, gray relational analysis was used to evaluate the safety of mine enterprises. Mine enterprise safety was ranked according to the gray relational degree, and weak links of mine safety practices identified based on gray relational analysis. Third, to validate the revised integrated weight method adopted in the process of gray relational analysis, the fuzzy evaluation method was used to the safety assessment of mine enterprises. Fourth, for first time, bow tie model was adopted to identify the causes and consequences of weak links and allow corresponding safety measures to be taken to guarantee the mine’s safe production. A case study of mine safety assessment was presented to demonstrate the effectiveness and rationality of the proposed composite risk analysis model, which can be applied to other related industries for safety evaluation.

## 1. Introduction

Mine production provides the necessary material foundation for the economic and social development of China [1–3], but, at the same time, it also causes many accidental deaths [4–6]. Therefore, mine safety plays an important role in protecting and fostering the rapid development of the national economy [7–9]. To prevent mine accidents, risk assessments first need to be performed.

Some scholars focus on a variety of risk assessment methods, such as the fuzzy evaluation method [10–12], neural network [13,14], set pair analysis [15–17], cloud model [18–20] and gray system theory [21,22]. Niknejad [10] had set up a fuzzy arithmetic for strategic risk management in global production networks, and the risk impacts on the inoperability of alternative global production network configurations, considering different risk scenarios, had been analyzed. Zhang [14] had established a comprehensive assessment model based on a fuzzy neural network to evaluate the environmental impact of mining areas, and the results showed that 7.5% of the studied regional mine sites were heavily damaged. Wang [15] had applied a set pair analysis for risk assessment for inrushing water, and the risk level of water intrusion in the Jigongling tunnel is high. Liu [19] had evaluated the stability of a rock slope based on a cloud model, and the most unfavorable factors were the behaviors of discontinuous materials, slope height and angle. Gray system theory is an uncertain method of studying the less data and poor information, especially suitable for data that are not easy to obtain [22]. Since the mine still belongs to the extensive production, some data are not easy to get, therefore gray system theory is applied to evaluate the mine safety. In the safety assessment fields, different assessment indicator weights might have influence on evaluation results [23–27]. Therefore, to determine the safety evaluation results exactly, assessment indicator weights first need to be calculated.

The methods for determining the weight of assessment indicators can be divided into the subjective, objective and integrated weight methods. The subjective weight method principally includes analytic hierarchy process [23] and Delphi method [24], which are mainly based on experts' subjective judgments rather than real data. The subjective weight method might take full advantage of the experts, but different evaluation results might be obtained from different experts. The objective weight method principally includes principal components analysis [25], entropy weight method [26] and variation coefficient method [27], which are mainly based on real data rather then expert judgments. The objective weight method uses the objectivity of real data but might not match the physical truth. The integrated weight method combines these two types of methods into one, which not only reflects expert judgments but also real data. In our intuitional understanding, the value of integrated weight should fall in between subjective and objective weights, but the results did not match in some reports [28–31], which will be discussed further below. To solve this problem, a new formula for integrated weight was adopted in this study.

After the assessment indicator weights were achieved, gray relational analysis was used to evaluate mine safety. Gray relational analysis is an important part of gray system theory [32], which has become very popular in many areas, such as green supplier selection [33], energy consumption, economic growth [34], and decision-making [35]. The relative importance of energy components on gross domestic product for Turkey had been obtained based on gray relational analysis, and the results showed that the most important energy sources for Turkey were oil and renewables [34].

The traditional safety assessment methods, such as the fuzzy evaluation method [10–12], neural network [13,14], set pair analysis [15–17] and cloud model [18–20], also including gray relational analysis [32], these methods can only identify the risk level and weak links of the evaluation object through the risk analysis, but cannot offer specific rectification measures for identified weak links. as they do not take advantage of evaluation results to promote effective, safe production. Therefore, an effective method needs to be found to analyze the weak links.

Bow tie model is also used widely as a risk analysis tool, which integrates basic causes, possible consequences, and corresponding safety measures of an accident in a transparent diagram. This model has been applied to many aspects of risk analysis, such as risk control [36], assessment [37], and management [38,39]. De Dianous [36] had applied bow tie model to risk control on site, and major accidents and barriers were identified using bow tie. Yazdi [39] had analyzed and estimated weak and strong points caused by H_2_S hazards based on the bow tie model. In this study, bow tie model was for the first time applied to analyze the weak links of mine safety practices.

The goal of this study was to build a composite risk analysis model for mine safety practices, using gray relational analysis and bow tie model and regard it as an extension to previous studies of grey relational analysis [40–42]. In addition, a new formula of integrated weight was adopted and, for the first time, the weak links of mine safety practices analyzed using the bow tie model.

This study was organized as follows: The fundamental theories of the composite risk analysis model were summarized in Section 2. The applicability of this composite risk analysis model was illustrated, using a case study of mine safety practices, in Section 3. Discussion of the results was presented in Section 4 and conclusions presented in Section 5.

## 2. Methods

Theoretical knowledge of the basic model of the variation coefficient method and gray relational analysis and bow tie model included in the present composite risk analysis model are presented in this section.

### 2.1. Gray relational analysis

Gray relational analysis is an important part of gray system theory [32], which is used to measure the gray relational degree between different factors. The greater the gray relational degree between two factors is, the greater the correlation degree. The less the gray relational degree between two factors is, the less the correlation degree. Calculation of the gray relational degree is the core of gray relational analysis and the process as described below.

Let *m* be the number of programs evaluated, *n* be the number of assessment indicators of each program evaluated, and the original data matrix of the programs evaluated is

$$A=\left[\begin{array}{llll} d_{1} & d_{2} & \cdots & d_{n}\\ a_{11} & a_{12} & \cdots & a_{1n}\\ \vdots & \vdots & & \vdots \\ a_{m1} & a_{m2} & \cdots & a_{mn} \end{array}\right]$$

where *a*_*ij*_ is the original data of the *j*th assessment indicator of the *i*th program evaluated.

The matrix *D* = [*d*_1_ *d*_2_ ⋯ *d*_*n*_] is the optimal index set, where *d*_*j*_ is the optimal value of the *j*th assessment indicator of programs evaluated. For the larger, the better assessment indicators, *d*_*j*_ is the maximum of the assessment indicators; for the smaller, the better assessment indicator, *d*_*j*_ is the minimum of the assessment indicators.

If all the assessment indicators of a program being evaluated reached their optimal state, the program evaluated was the optimal index set and the best program. However, in reality, assessment indicators of each program evaluated did not reach optimal values at the same time and gray relational analysis was then used to evaluate the programs.

In the evaluation process, due to the different dimensions of assessment indicators, differences in numerical values in the original data might be large, such that gray relational analysis cannot be directly applied. Therefore, the assessment indicators needed to be rendered dimensionless. Here, a mean transformation was applied to nondimensionalize the assessment indicators, using formulas 1 and 2:

$$\bar{a_{j}}=\frac{\sum_{i=1}^{m}a_{ij}}{m}\;j=1,2,\cdots ,n$$ (1)

$$b_{ij}=\frac{a_{ij}}{\bar{a_{j}}}$$ (2)

After yielding assessment indicators dimensionless, the original data matrix was transferred

$$B=\left[\begin{array}{llll} b_{11} & b_{12} & \cdots & b_{1n}\\ b_{21} & b_{22} & \cdots & b_{2n}\\ \vdots & \vdots & & \vdots \\ b_{m1} & b_{m2} & \cdots & b_{mn} \end{array}\right]$$

The optimal index set was transfered into D* = [*d*_1_* *d*_2_* ⋯ *d*_n_*]. Then, with the optimal index set D* the reference sequence and *B* the sequence compared, the gray relational coefficient of the *j*th assessment indicator of the *i*th program evaluated was achieved according to gray relational analysis, and formula 3 was

$$\xi_{ij}=\frac{\underset{1\leq j\leq n}{\underset{1\leq i\leq m}{\min}}\left|d_{j}{}^{*}-b_{ij}\right|+\rho \underset{1\leq j\leq n}{\underset{1\leq i\leq m}{\max}}\left|d_{j}{}^{*}-b_{ij}\right|}{\left|d_{j}{}^{*}-b_{ij}\right|+\rho \underset{1\leq j\leq n}{\underset{1\leq i\leq m}{\max}}\left|d_{j}{}^{*}-b_{ij}\right|}$$ (3)

The significant differences between the gray relational coefficients was improved by weakening the influence on the data, due to the maximum absolute value being too large, through the addition of the resolution coefficient *ρ* in formula 3 and usually set to *ρ* = 0.5.

Let the weights of assessment indicators of the programs be evaluated by *W* = [*w*_1_,*w*_2_,⋯,*w*_*n*_].

Thus far, the gray relational degree of programs evaluated was achieved according to formula 4:

$$r_{i}=\sum_{j=1}^{n}\xi_{ij}\times w_{j}\;i=1,2,\cdots ,m$$ (4)

The larger the gray relational degree *r*_*i*_, the closer the *i*th program evaluated was to the optimal index set D*. Accordingly, the order of the programs evaluated was determined.

### 2.2. Variation coefficient method

The gray relational degree of the programs evaluated was achieved by first determining the assessment indicator weights. In determining these weights, the evaluation results deviated because of the influence of subjective factors in the subjective weight method. The variation coefficient method is an objective weight method [27], in which the weight is obtained based on original data, reflecting objective changes in the index information. A larger variation degree in the assessment indicators indicates that the programs evaluated have good uniqueness in this regard and, thus, a larger weight should be given to these assessment indicators; otherwise, a smaller weight should be given to the indicators. The process of calculating assessment indicator weights according to variation coefficient method is described below.

The expectation and standard deviation of assessment indicators were calculated based on formulas 5 and 6:

$$\mu_{j}=\frac{1}{m}\sum_{i=1}^{m}a_{ij}\;j=1,2,\cdots ,n$$ (5)

$$\sigma_{j}=\sqrt{\frac{1}{m}\sum_{i=1}^{m}{\left(a_{ij}-\mu_{j}\right)}^{2}}$$ (6)

The variation coefficient of assessment indicators was achieved based on formula 7:

$$\delta_{j}=\frac{\sigma_{j}}{\mu_{j}}$$ (7)

The weight of assessment indicators was obtained by normalization of the variation coefficient based on formula 8:

$$w_{j}=\frac{\delta_{j}}{\sum_{j=1}^{n}\delta_{j}}$$ (8)

### 2.3. Bow tie model

**Fig 1:**
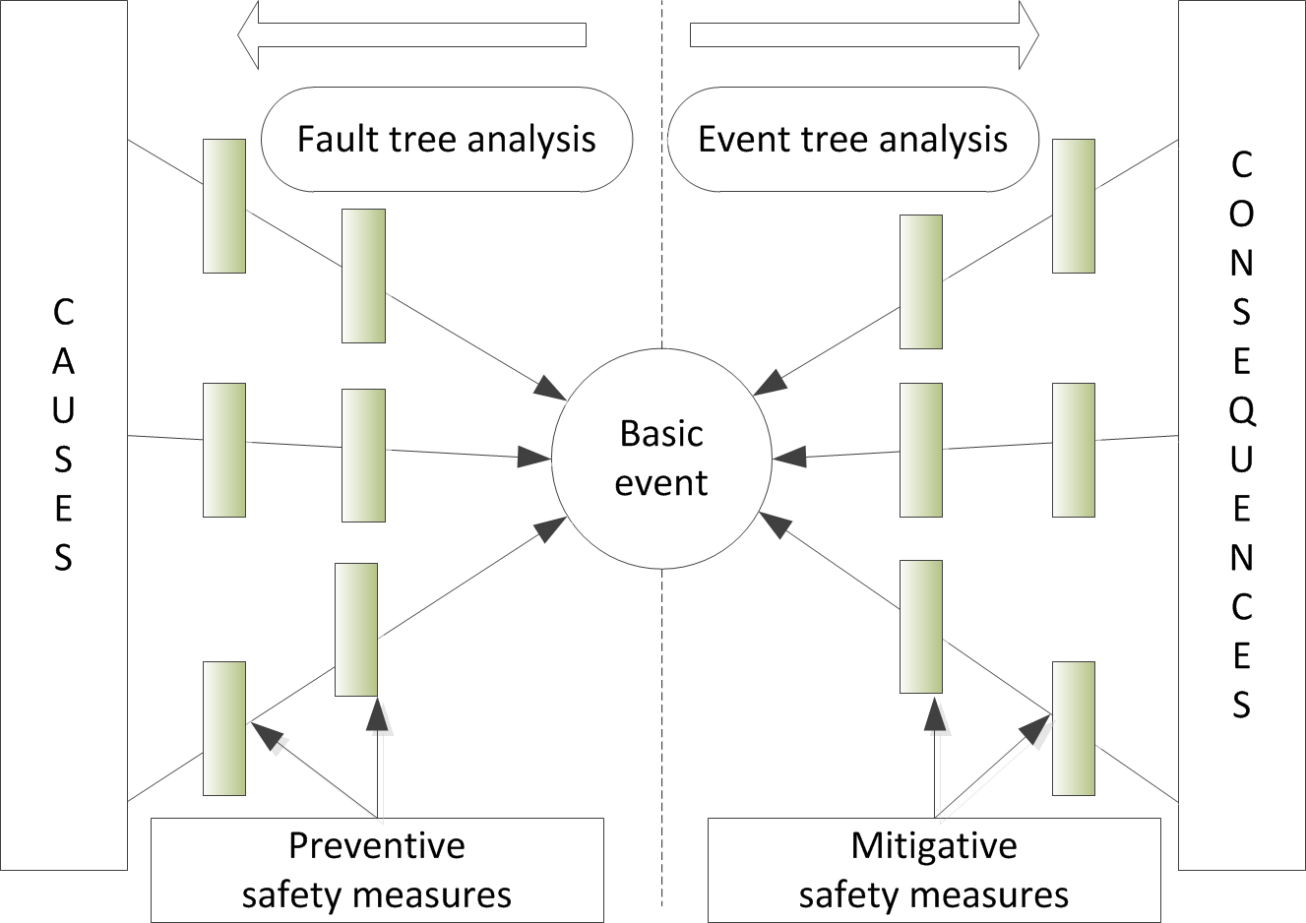
Bow tie model.

Bow tie model is a safety assessment method that consists of a fault tree on the left side and an event tree on the right side and centered in it is a basic event (Fig 1). The causes of an event are indicated on the left of the bow tie and consequences on the right. The causes are the events that might lead to accidents and the consequences are the losses (including health and property) through accidents. To prevent basic accidents, safety barriers should be adopted. Preventive safety measures are set on the fault side and, thus, come before the basic event. At the same time, mitigative safety measures are set on the event tree side and, thus, come after the basic event.

## 3. Results

Assessment indicator weights are first calculated based on the variation coefficient method in this section and, then, gray relational analysis applied to evaluate mine safety practices.

### 3.1. Application

The primary critical factors in mine can be divided as follows according to the classification for casualty accidents of enterprise staff and workers: object strike, mechanical injury, electric shock, crashing from the high, roof fall and wall collapse, blasting, gas explosion, poisoning and asphyxia, vehicle injury, crane injury, fire hazard, collapse, permeable, gunpowder explosion and so on.

The above accidents can cause serious injury and death, and the accidental injury and death rate can best reflect mine safety practices and is affected by various factors, mainly including staff training rate, post variation rate, safety management ability, and safety input from the safety management perspective. The staff training rate is the proportion of the number of staff who received job training to the total number of staff enrolled within the same year; post variation rate is the proportion of the number of staff who changed the post to the total number of staff enrolled within the same year; safety management ability refers to the knowledge and ability that safety administration should have, also including that whether the safety management organization and the rules and regulations are perfect, which can be determined according to the experts' scoring; safety input includes security costs, staff training costs and so on. Four mine enterprises [40] have been chosen for program evaluation and their basic information shown in Table 1.

**Table 1:** Basic information of mine enterprises.

Mine enterprises	Staff training rate	Post variation rate	Safety management ability	Safety input
MA	0.18	0.014	80	14.5
MB	0.14	0.014	86	22
MC	0.15	0.0029	94	10
MD	0.17	0.0036	96	9

where the unit of safety input is ten thousand yuan.

### 3.2. Computational process and results

The staff training rate, safety management ability, and safety input were the larger and, clearly, the better assessment indicators (Table 1). The assessment indicator of the post variation rate indicated both positive and negative impacts on mine enterprises. A reasonable and appropriate post variation rate can introduce new ideas and thoughts for an enterprise, thus preventing an inherent management model and rigidity of thinking, are conductive for maintaining enterprise vitality. Managers can reallocate staff work according to employee characteristics and outstanding staff can be absorbed to pursue creative work. From these points of view, the post variation rate should be the larger, the better assessment indicator. However, when the post variation rate is beyond normal range, the normal operation of enterprises is affected and management costs increased, resulting in negative effects. To be consistent with the original paper [40] and more easily compare the present evaluation results, here, the post variation rate was considered as the smaller but better assessment indicator. The original data matrix was shown as follows according to Table 1

$$A=\left[\begin{array}{llll} 0.18 & 0.0029 & 96 & 22\\ 0.18 & 0.014 & 80 & 14.5\\ 0.14 & 0.014 & 86 & 22\\ 0.15 & 0.0029 & 94 & 10\\ 0.17 & 0.0036 & 96 & 9 \end{array}\right]$$

The expectation, standard deviation, variation coefficient, and assessment indicator weights were shown in Table 2 according to formulas 5–8.

**Table 2:** Weight calculated with the variation coefficient method.

Process	Staff training rate	Post variation rate	Safety management ability	Safety input
Expectation	0.16	0.0086	89	13.875
Standard deviation	0.0158	0.0054	6.4031	5.128
Variation coefficient	0.0988	0.6279	0.0719	0.3696
Weight	0.0846	0.5375	0.0615	0.3164

A nondimensionalized original data matrix of assessment indicators follows according to formulas 1 and 2.

$$B=\left[\begin{array}{llll} 1.125 & 0.3372 & 1.0787 & 1.5856\\ 1.125 & 1.6279 & 0.8989 & 1.045\\ 0.875 & 1.6279 & 0.9663 & 1.5856\\ 0.9375 & 0.3372 & 1.0562 & 0.7207\\ 1.0625 & 0.4186 & 1.0787 & 0.6486 \end{array}\right]$$

Now, the optimal index set was D* = [1.125 0.3372 1.0787 1.5856]. The gray relational coefficient was obtained according to formula 3

$$E=\left[\begin{array}{cccc} 1 & 0.3333 & 0.7821 & 0.5442\\ 0.7208 & 0.3333 & 0.8517 & 1\\ 0.7749 & 1 & 0.9663 & 0.4273\\ 0.9117 & 0.888 & 1 & 0.4078 \end{array}\right]$$

The gray relational degrees of mine enterprises were obtained based on the gray relational coefficient and assessment indicator weights according to formula 4

$$R=E\times W=\left[\begin{array}{cccc} 1 & 0.3333 & 0.7821 & 0.5442\\ 0.7208 & 0.3333 & 0.8517 & 1\\ 0.7749 & 1 & 0.9663 & 0.4273\\ 0.9117 & 0.888 & 1 & 0.4078 \end{array}\right]\times \left[\begin{array}{l} 0.0846\\ 0.5375\\ 0.0615\\ 0.3164 \end{array}\right]=\left[\begin{array}{l} 0.484\\ 0.609\\ 0.798\\ 0.745 \end{array}\right]$$

According to the gray relational degree, mine safety from high to low was MC, MD, MB and MA, respectively.

## 4. Discussion

### 4.1. Weight determined by other methods

#### 4.1.1. Delphi method

Assessment indicator weights were determined by Delphi method in the original paper [40]. Delphi method is inviting experts in related fields to make comments on certain issues. Then the views of experts are scientifically integrated, collated and summarized, anonymously feedback the results to each expert to consult again. After many rounds of consult until the views of experts tend to be focused, getting a more consistent and reliable opinion. The assessment indicator weight determined by Delphi method was: [staff training rate, post variation rate, safety management ability, safety input] = [0.24, 0.19, 0.31, 0.26], and the gray relational degree of mine enterprises in the original paper [40] was: [MA, MB, MC, MD] = [0.710, 0.774, 0.812, 0.820], and mine safety from high to low was MD, MC, MB, and MA.

Comparison of evaluation results from the two different methods were observed not to be consistent. MD has been evaluated to have the highest safety, followed by MC, based on the subjective weight method in the original paper [40]. Here, MC was evaluated to have the highest safety, followed by MD, based on the objective weight method. The main reason for this difference was different weight assignments of assessment indicators. For example, the weight of post variation rate was the largest based on the objective weight method, while it was the smallest based on the subjective weight method. In addition, the original paper did not compare evaluation results with the actual safety of mine enterprises. Therefore, to reduce the error caused by the subjective and objective weight methods in evaluation results, it was necessary to combine the two methods together to evaluate mine enterprise safety.

#### 4.1.2. Traditional integrated weight method

Integrated weight formula 9 is usually used in these studies [28–31].

$$\lambda_{i}=\frac{w_{i}\theta_{i}}{\sum_{i=1}^{n}w_{i}\theta_{i}}\;i=1,2,\cdots ,n$$ (9)

where *w*_*i*_ is the subjective weight of the *i*th assessment indicator, *θ*_*i*_ the objective weight of the *i*th assessment indicator, *λ*_*i*_ the integrated weight of the *i*th assessment indicator, and *n* the number of assessment indicators.

There is a problem with the traditional integrated weight calculated by formula 9. If the subjective weight was not equal to the objective weight, the integrated weight should fall in between the subjective and objective weight. In particular, if the subjective and objective weights were equal, the integrated weight should be equal to these weights. However, the integrated weight calculated by formula 9 was outside the range of the subjective to objective weight or objective to subjective weight. For example, one program that was evaluated had three assessment indicators, with subjective weights at *w*_1_ = 0.5, *w*_2_ = 0.3 and *w*_3_ = 0.2 and the objective weights at *θ*_1_ = 0.6, *θ*_2_ = 0.15 and *θ*_3_ = 0.25. The reasonable integrated weights of assessment indicators should have been *λ*_1_ ∈ [0.5, 0.6], *λ*_2_ ∈ [0.15, 0.3] and *λ*_3_ ∈ [0.2, 0.25]. However, the integrated weights calculated by formula 9 were *λ*_1_ = 0.76, *λ*_2_ = 0.11 and *λ*_3_ = 0.13, all outside of the ranges between subjective and objective weights.

#### 4.1.3. Revised integrated weight method

To solve this problem, the formula for calculating integrated weight was revised as follow.

$$\lambda_{i}=\delta w_{i}+\left(1-\delta \right)\theta_{i}\;i=1,2,\cdots ,n$$ (10)

where *δ* ∈ [0,1] is the preference coefficient, with *δ* → 0 indicating that the integrated weight was mainly determined by the objective weight, and *δ* → 1 indicating that the integrated weight was mainly determined by the subjective weight. In practical application, if *δ* = 0.5, the integrated weight was in the range expected from the subjective and objective weights. Thus, the formula for calculating integrated weight was transformed into formula 11 [43]:

$$\lambda_{i}=\frac{w_{i}+\theta_{i}}{2}$$ (11)

The assessment indicator weights determined by subjective Delphi method and objective variation coefficient method were: [staff training rate, post variation rate, safety management ability, safety input] = [0.24, 0.19, 0.31, 0.26] and [0.0846, 0.5375, 0.0615, 0.3164] respectively. Therefore, the integrated weight was achieved based on the subjective and objective weights according to formula 11, and the result was: [staff training rate, post variation rate, safety management ability, safety input] = [0.1623, 0.3638, 0.1857, 0.2882]. The gray relational degree of mine enterprises was: [MA, MB, MC, MD] = [0.586, 0.685, 0.792, 0.774]. The evaluation results based on revised integrated weights also indicated MC as having the highest safety, followed by MD, but the difference value of gray relational degree between MC and MD was smaller than with the objective variation coefficient method. Therefore, it was concluded that the evaluation result had a deviation in terms of the actual safety of mine enterprises in the original paper [40].

### 4.2. Compare with fuzzy evaluation method

To validate the revised integrated weight method adopted in the process of gray relational analysis, this section compare the results of safety assessment with fuzzy evaluation method [44]. The brief procedure of fuzzy evaluation method was as follows.

The nondimensionalize of the original data is calculated based on formulas 12 and 13.

$$c_{ij}^{s}=\frac{\underset{1\leq i\leq m}{\min}a_{ij}}{a_{ij}}\;j=1,2,\cdots ,n$$ (12)

$$c_{ij}^{l}=\frac{a_{ij}}{\underset{1\leq i\leq m}{\max}a_{ij}}\;j=1,2,\cdots ,n$$ (13)

where formula 12 is used for the smaller the better assessment indicators; formula 13 is used for the larger the better assessment indicators.

After nondimensionalize, the membership of mine enterprises can be calculated based on the dimensionless data and revised integrated weight as follow.

$$F=\left[\begin{array}{cccc} 1 & 0.2071 & 0.8333 & 0.6591\\ 0.7778 & 0.2071 & 0.8958 & 1\\ 0.8333 & 1 & 0.9792 & 0.4545\\ 0.9444 & 0.8056 & 1 & 0.4091 \end{array}\right]\times \left[\begin{array}{l} 0.1623\\ 0.3638\\ 0.1857\\ 0.2882 \end{array}\right]=\left[\begin{array}{l} 0.5823\\ 0.6561\\ 0.8119\\ 0.75 \end{array}\right]$$

According to the fuzzy evaluation method, mine safety from high to low was MC, MD, MB and MA, respectively.

The safety assessment results of mine enterprises using fuzzy evaluation method were the same to the gray relational analysis, in which the assessment indicators weights were determined by revised integrated weight method, proved that the safety assessment method proposed in this paper was feasible.

### 4.3. Bow tie analysis of weak links

**Fig 2:**
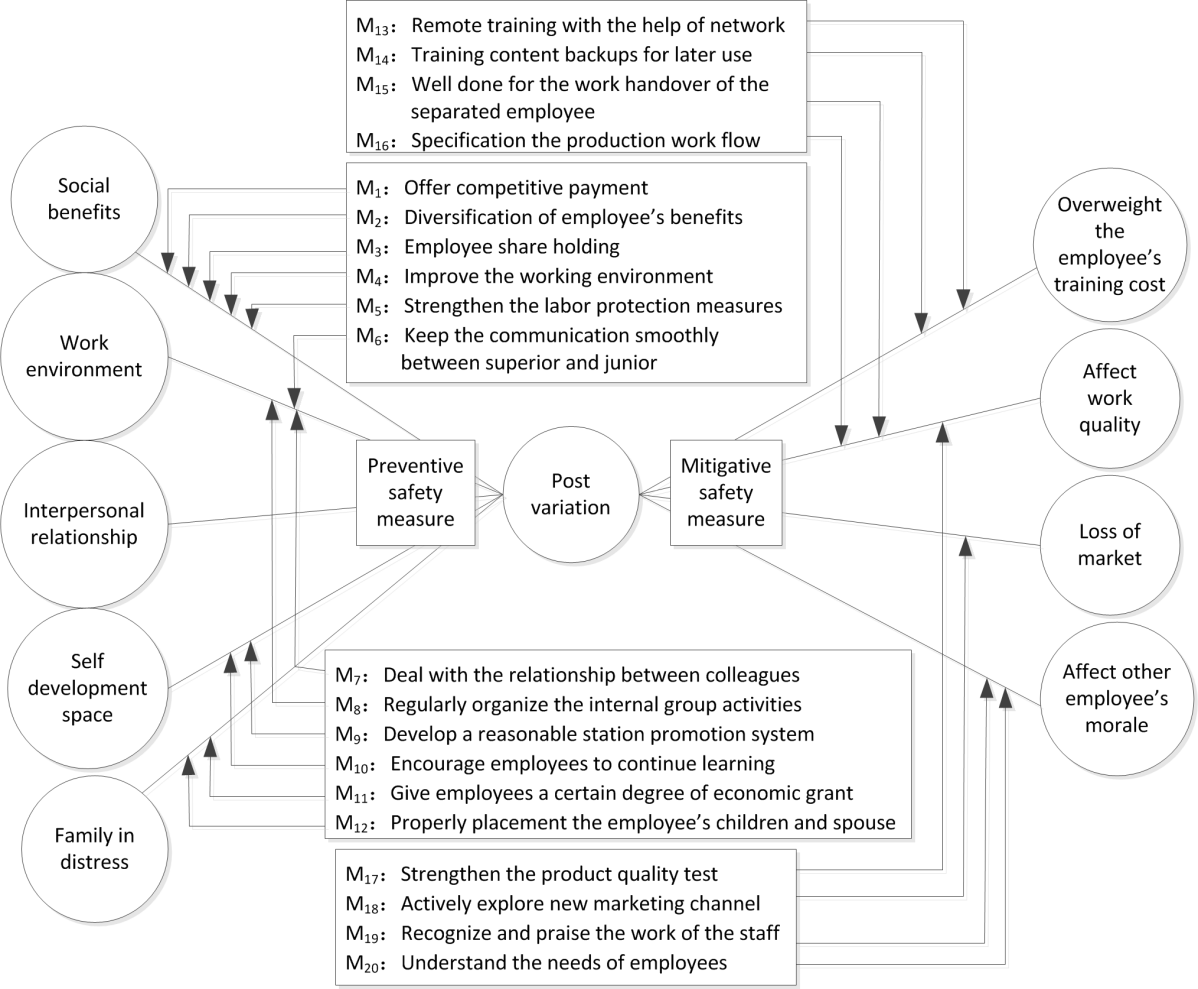
Bow tie analysis of post variation.

It can be seen from the above analysis that evaluation results based on the objective and integrated weight methods were not consistent with the subjective weight method, mainly because the weight of the post variation rate was not assigned in the same manner. Post variation is an important factor affecting mine safety, having great influence on evaluation results. In previous studies, only a simple analysis of the identified weak links have been carried out after the safety evaluation of mine enterprises, which failed to identify the causes and consequences of the weak links and could not effectively improve the safety situation of these enterprises [40–42]. Therefore, post variation was taken as the basic event for bow tie analysis (Fig 2), identifying the causes and consequences led by post variation and taking corresponding safety measures to prevent the basic event.

The left of a bow tie was a fault tree analysis that included five causes that can lead to post variation and the right of the bow tie was an event tree analysis that included four consequences of post variation (Fig 2). Twelve preventive safety measures were set on the left to prevent the occurrence of post variation and eight mitigative safety measures set on the right to mitigate the consequences of post variation. The risk of post variation was thus further reduced by this bow tie analysis.

### 4.4. Brief summary of discussion

The present results confirmed that the composite risk analysis model proposed here was successfully applied to the assessment of mine safety practices, in which a new formula for integrated weight was adopted and identified weak links analyzed by bow tie analysis for the first time. The advantages of the composite risk analysis model proposed in this paper were as follows. First, the safety of mines was evaluated so as to compare the safety of different mines. Second, the objective variation coefficient method was first applied to calculate the weight of assessment indicators, avoiding the interference of human factors and also saving a lot of manpower. Third, the bow tie analysis can identify the causes and consequences of the weak links, which is easy to understand the critical event in-depth and provide reasonable suggestions for the safe production of mine. Also, to validate the revised integrated weight method adopted in the process of gray relational analysis, the fuzzy evaluation method was used to the safety assessment of mine enterprises.

Motivated by the previous studies on gray relational analysis [32–35] and bow tie model [36–39], these two methods were used for the safety assessment of mine for the first time. In order to eliminate the influence of human factors, such as subjective weight method, the objective variation coefficient method [27] was adopted in the process of gray relational analysis, which also saves a lot of manpower. In previous studies [28–31], formula 9 for calculating integrated weight had a problem in which the integrated weight was outside the ranges of paired subjective and objective weights. To solve this problem, an expectation formula that ameliorated this problem was adopted. In addition, only a simple analysis of the identified weak links have been carried out after the safety evaluation of mine enterprises [40–42], which failed to identify the causes and consequences of the weak links and could not effectively improve the safety situation of these enterprises. Therefore, post variation was taken as the basic event for bow tie analysis for the first time, identifying the causes and consequences led by post variation and taking corresponding safety measures to prevent the basic event. The approach proposed in this paper can be applied to other related industries for safety evaluations.

To simplify the discussion, the perference coefficient *δ* in formula 10 was chosen as only 0.5. Future research should focus on the influence of this preference coefficient in safety evaluation results.

## 5. Conclusion

A composite risk analysis model of mine safety practices based on gray relational analysis and bow tie model was proposed. The main conclusions were as follows.

First, assessment indicator weights were determined by the objective variation coefficient method, and the safety evaluation results for mine enterprises were obtained based on gray relational analysis, mine safety from high to low was MC, MD, MB and MA, respectively.

Second, a new formula was adopted to calculate the integrated weights, and mine safety from high to low was also MC, MD, MB and MA. The evaluation results based on integrated weights also indicated MC as having the highest safety, followed by MD, but the difference value of gray relational degree between MC and MD was smaller than with the objective weight method.

Third, to validate the revised integrated weight method adopted in the process of gray relational analysis, the fuzzy evaluation method was used to the safety assessment of mine enterprises. Both methods had the same safety assessment results, showed that the safety assessment method proposed in this paper was feasible.

Fourth, the identified weak link post variation was analyzed using bow tie model for the first time, in which twelve preventive safety measures were set on the left to prevent the occurrence of post variation and eight mitigative safety measures set on the right to mitigate the consequences of post variation. The risk of post variation was thus further reduced by this bow tie analysis.
